# Cimetidine and therapy of rodent tumours.

**DOI:** 10.1038/bjc.1982.98

**Published:** 1982-04

**Authors:** D. Hannant, K. James, R. E. Bolton, M. D. Robertson, I. Milne


*Br. J. Cancer* (1982) **45**, 613

***Short Communication***

### CIMETIDINE AND THERAPY OF RODENT TUMOURS

D. HANNANT, K. JAMES*, R. E. BOLTON, M. D. ROBERTSON AND I. MILNE*

*From the Institute of Occupational Medicine, Roxburgh Place, Edinburgh, EH8 9SU and the *Department of Surgery, University of Edinburgh, Teviot Place, Edinburgh EH8 9AG*

Received 9 November 1981 Accepted 6 January 1982

A NUMBER of recent studies have shown that the growth of certain rodent tumours may be inhibited by the oral administration of the H-2 antagonist cimetidine (Gifford *et al.*, 1981; Osband *et al.*, 1981). Furthermore, it has been proposed that the antitumour effects of this compound are due to its ability to inhibit histamine-induced T-suppressor-cell activation (Osband *et al.*, 1980*a*, 1981; Ogden & Hill, 1980; Gifford *et al.*, 1981). In view of the potential of this approach to immunotherapy we decided to undertake studies on the effect of similar cimetidine protocols on the growth of some of the experimental rodent tumours routinely used in our laboratories. The results of these studies are summarized below.

Male syngeneic WAG/Ed (Edinburgh University Centre for Laboratory Animals strain of Wistar rats) were inoculated s.c. with $10^5$ viable cells of the asbestos-induced mesothelioma MF3 (Bolton *et al.*, in preparation) and cimetidine (Smith, Kline and French Laboratories Ltd., Welwyn Garden City) was included in the drinking water from the date of tumour inoculation. Similarly, $10^5$ or $10^6$ viable cells of the 3-methylcholanthrene (MCA)-induced fibrosarcoma CCH1 (Woodruff *et al.*, 1972) were inoculated s.c. into syngeneic CBA/Ca mice, and cimetidine included in the drinking water. In both experiments, the concentrations of cimetidine in the drinking water were such that each animal received 100 mg/kg/day from the day of tumour inoculation. Daily water consumption was recorded for all animals on a cage basis for several days before experiments to calculate the mean volume of water imbibed by each mouse or rat. Control animals were inoculated with tumour but received unadulterated drinking water. Each cage contained 4 to 5 animals. Tumour diameters were measured at regular intervals with vernier-scale calipers.

Spleen cells were prepared from syngeneic WAG/Ed rats, and the effect of cimetidine on the *in vitro* binding of histamine to spleen lymphocytes was followed as described by Osband *et al.* (1980*b*). Histamine dihydrochloride (Sigma, Poole) was coupled to fluorescinated bovine serum albumin (B.S.A., Sigma) with 1-ethyl 3 (3-dimethylamino-propyl) carbodiimide hydrochloride (Sigma) at pH 5·6 using a method modified from Hannant *et al.* (1980).

The results of some of the *in vivo* experiments are summarized in the Table. They show that, at the concentration of cimetidine used, there was no observable effect on the incidence or size of tumour in the rat and mouse models. The dose of cimetidine used was the same as that shown by Gifford *et al.* (1981) to have a maximum inhibitory effect on the growth of a syngeneic MCA-induced fibrosarcoma in C3H mice and a lymphoma ascites of C57BL/6 mice. The MCA-induced fibrosarcoma used in our study was syngeneic

Correspondence to: Duncan Hannant, Medical Branch, Institute of Occupational Medicine, Roxburgh Place, Edinburgh EH8 9SU.

TABLE—*Effect of oral cimetidine on the growth of rodent tumours**

| Treatment group | Mean tumour‡ diameter (mm ± s.d.) | No. animals with tumour | Days after tumour inoculation |
|---|---|---|---|
| CCH1 control | 20·7 ± 2·6 | 10/10 | 29 |
| CCH1 cimetidine† | 19·75 ± 2·9 | 10/10 | |
| MF3 | 23·2 ± 3·0 | 5/8 | 38 |
| MF3 cimetidine | 23·7 ± 10·0 | 5/8 | |

* Rats challenged with $10^5$ cells; mice with $10^6$ cells. Similar results, however, were obtained in mice challenged with $10^5$ cells.

† Cimetidine was included in the drinking water to give 100 mg/kg/day to each animal. It was assumed that the fluid intake by each animal in the cage was similar. While this assumption is not entirely satisfactory, within-group differences in tumour growth which might be attributed to differences in fluid intake of individual mice were not obvious, as in most groups the growth rates were very constant.

‡ No difference in the growth rates of tumours between test and control groups was observed.

to CBA mice, and whilst the use of a different mouse strain might have had some effect, the complete absence of any tumour inhibition is unlikely to be a result of sub-optimal cimetidine concentration. Other studies (results not presented) with tumour CCH1 showed that concentrations of cimetidine up to 200 mg/kg/day also had no observable effect. In contrast, Gifford *et al.* (1981) found that tumour growth in mice was sensitive to cimetidine concentrations ranging from 15 to 200 mg/kg/day.

The lack of effect in the rat mesothelioma experiments is not because cimetidine does not function as a H-2-receptor antagonist in this species. *In vitro* studies clearly demonstrated that histamine (labelled with fluorescinated BSA) was able to bind specifically to 33·9 ± 8·8% (mean ± s.d.) of normal WAG/Ed rat spleen cells. Pretreatment of these cells with $10^{-3}$M cimetidine for 1 h at 37°C before washing and incubation with the fluorescence reagent reduced the fluorescent staining to 12·1 ± 2·7%. Therefore, although cimetidine failed to influence tumour growth, it did bind *in vitro* to H-2 receptors on rat spleen cells.

In summary, cimetidine has been shown to function as an H-2 receptor antagonist in both mice and rats, and to be an effective immunostimulator in therapy of some tumours. However, it appears that the compound may not have universal application in tumour therapy, because it can exhibit variable effects on tumours within the same species (mouse) and antitumour effects in tumour-bearing rats have yet to be demonstrated. Moreover, a recent report suggests that cimetidine has no effect on immunological parameters in man (Festen *et al.*, 1981). In the light of these observations we would suggest that further studies with this compound are necessary before any decision can be reached on its suitability for clinical use.

This work was supported in part by a grant awarded to Dr Keith James from the Cancer Research Campaign.

REFERENCES

FESTEN, H. P. M., DE PAUW, B. E., SMEULDERS, J. & WAGENER, D. J. (1981) Cimetidine does not influence immunological parameters in man. *Clin. Immunol. Immunopathol.*, **21**, 33.

GIFFORD, R. R. M., FERGUSON, R. M. & VOSS, B. V. (1981) Cimetidine reduction of tumour-formation in mice. *Lancet*, i, 638.

HANNANT, D., BOWEN, J. G., PRICE, M. R. & BALDWIN, R. W. (1980) Radio-iodination of rat hepatoma-specific antigens and retention of serological reactivity. *Br. J. Cancer*, **41**, 716.

OGDEN, B. E. & HILL, R. E. (1980) Histamine regulates lymphocyte mitogenic response through activation of specific H1 and H2 histamine receptors. *Immunology*, **41**, 107.

OSBAND, M. E., GALLISON, D., MILLER, B., AGARWAL, R. P. & MCCAFFREY, R. P. (1980*a*) Concanavalin A activation of suppressor cells is mediated by the release of histamine and is blocked by cimetidine. *Clin. Res.*, **28**, 356a.

OSBAND, M. E., COHEN, E. B., MCCAFFREY, R. P. & SHAPIRO, H. M. (1980*b*) A technique for the flow cytometric analysis of lymphocytes bearing histamine receptors. *Blood*, **65**, 923.

OSBAND, M. E., SHEN, Y.-J., SCHLESINGER, M. & 5 others (1981) Successful tumour immunotherapy with cimetidine in mice. *Lancet*, i, 636.

WOODRUFF, M. F. A., INCHLEY, M. P. & DUNBAR, N. (1972) Further observations on the effect of *C. parvum* and anti-tumour globulin on syngeneically transplanted mouse tumours. *Br. J. Cancer*, **26**, 67.


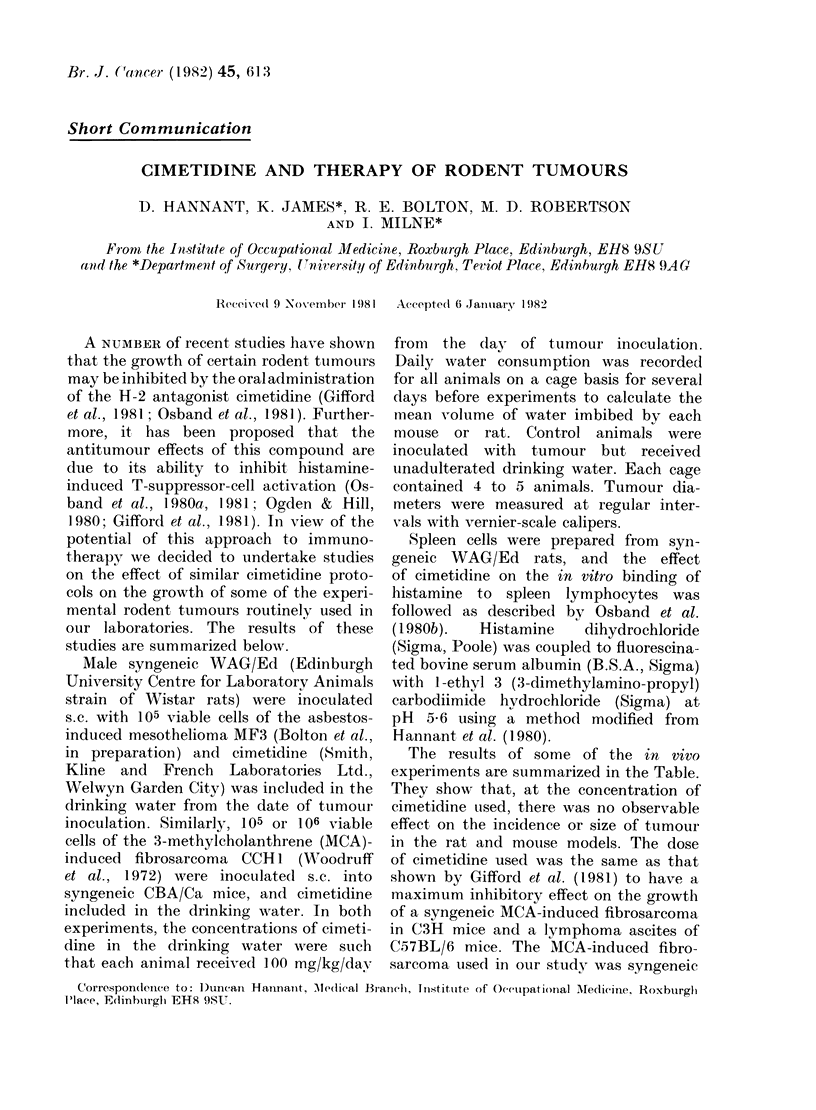

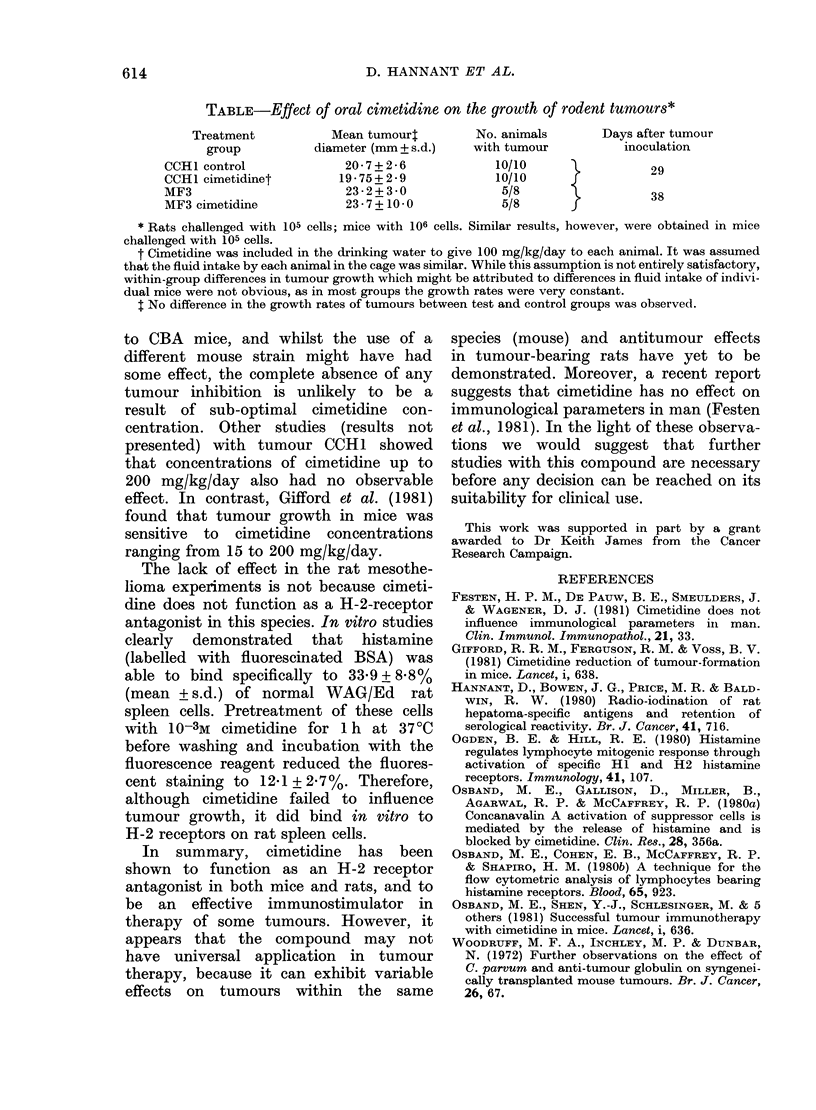


## References

[OCR_00214] Gifford R. R., Ferguson R. M., Voss B. V. (1981). Cimetidine reduction of tumour formation in mice.. Lancet.

[OCR_00221] Hannant D., Bowen J. G., Price M. R., Baldwin R. W. (1980). Radioiodination of rat hepatoma-specific antigens and retention of serological reactivity.. Br J Cancer.

[OCR_00225] Ogden B. E., Hill H. R. (1980). Histamine regulates lymphocyte mitogenic responses through activation of specific H1 and H2 histamine receptors.. Immunology.

[OCR_00238] Osband M. E., Cohen E. B., McCaffrey R. P., Shapiro H. M. (1980). A technique for the flow cytometric analysis of lymphocytes bearing histamine receptors.. Blood.

[OCR_00244] Osband M. E., Hamilton D., Shen Y. J., Cohen E., Shlesinger M., Lavin P., Brown A., McCaffrey R. (1981). Successful tumour immunotherapy with cimetidine in mice.. Lancet.

[OCR_00249] Woodruff M. F., Inchley M. P., Dunbar N. (1972). Further observations on the effect of C. parvum and anti-tumour globulin on syngeneically transplanted mouse tumours.. Br J Cancer.

[OCR_00208] ten Berge R. J., de Pauw B. E., Smeulders J., Wagener D. J. (1981). Cimetidine does not influence immunological parameters in man.. Clin Immunol Immunopathol.

